# Supplementary material for: Resolving the full spectrum of human genome variation using Linked-Reads
Source: Genome Res. 2019 Apr;29(4):635–45. doi: 10.1101/gr.234443.118 (PMC6442396; doi:10.1101/gr.234443.118)
Supplement: Supplemental Material [file supp_gr.234443.118_Supplemental_longranger-2.2.zip › longranger-2.2.tar.bz2!/lr2.2-public/lib/stan/cmdstan/stan/doc/api/html/var__decls__grammar_8hpp_source.html]

Stan: src/stan/gm/grammars/var\_decls\_grammar.hpp Source File


|  |  |
| --- | --- |
|  | Stan  2.5.0  probability, sampling & optimization |


- Main Page
- Related Pages
- Namespaces
- Classes
- Files

- File List
- File Members

All Classes Namespaces Files Functions Variables Typedefs Enumerations Enumerator Friends Macros Pages

- src
- stan
- gm
- grammars

var\_decls\_grammar.hpp

Go to the documentation of this file.

1 #ifndef STAN\_\_GM\_\_PARSER\_\_VAR\_DECLS\_GRAMMAR\_\_HPP

2 #define STAN\_\_GM\_\_PARSER\_\_VAR\_DECLS\_GRAMMAR\_\_HPP

3

4 #include <string>

5 #include <sstream>

6 #include <vector>

7

8 #include <boost/spirit/include/qi.hpp>

9

10 #include <stan/gm/ast.hpp>

11 #include <stan/gm/grammars/whitespace\_grammar.hpp>

12 #include <stan/gm/grammars/expression\_grammar.hpp>

13 #include <stan/gm/grammars/expression07\_grammar.hpp>

14

15 namespace stan {

16

17  namespace gm {

18

19  template <typename Iterator>

20  struct var\_decls\_grammar

21  : boost::spirit::qi::grammar<Iterator,

22  boost::spirit::qi::locals<bool>,

23  std::vector<var\_decl>(bool,var\_origin),

24  whitespace\_grammar<Iterator> > {

25

26  var\_decls\_grammar(variable\_map& var\_map,

27  std::stringstream& error\_msgs);

28

29  // global info for parses

30  variable\_map& var\_map\_;

31  std::stringstream& error\_msgs\_;

32

33  // grammars

34  expression\_grammar<Iterator> expression\_g;

35

36  expression07\_grammar<Iterator> expression07\_g;

37

38  // rules

39

40  boost::spirit::qi::rule<Iterator,

41  cholesky\_factor\_var\_decl(var\_origin),

42  whitespace\_grammar<Iterator> >

43  cholesky\_factor\_decl\_r;

44

45  boost::spirit::qi::rule<Iterator,

46  cholesky\_corr\_var\_decl(var\_origin),

47  whitespace\_grammar<Iterator> >

48  cholesky\_corr\_decl\_r;

49

50  boost::spirit::qi::rule<Iterator,

51  corr\_matrix\_var\_decl(var\_origin),

52  whitespace\_grammar<Iterator> >

53  corr\_matrix\_decl\_r;

54

55  boost::spirit::qi::rule<Iterator,

56  cov\_matrix\_var\_decl(var\_origin),

57  whitespace\_grammar<Iterator> >

58  cov\_matrix\_decl\_r;

59

60  boost::spirit::qi::rule<Iterator,

61  std::vector<expression>(var\_origin),

62  whitespace\_grammar<Iterator> >

63  dims\_r;

64

65  boost::spirit::qi::rule<Iterator,

66  double\_var\_decl(var\_origin),

67  whitespace\_grammar<Iterator> >

68  double\_decl\_r;

69

70  boost::spirit::qi::rule<Iterator,

71  std::string(),

72  whitespace\_grammar<Iterator> >

73  identifier\_r;

74

75  boost::spirit::qi::rule<Iterator,

76  std::string(),

77  whitespace\_grammar<Iterator> >

78  identifier\_name\_r;

79

80  boost::spirit::qi::rule<Iterator,

81  int\_var\_decl(var\_origin),

82  whitespace\_grammar<Iterator> >

83  int\_decl\_r;

84

85  boost::spirit::qi::rule<Iterator,

86  matrix\_var\_decl(var\_origin),

87  whitespace\_grammar<Iterator> >

88  matrix\_decl\_r;

89

90  boost::spirit::qi::rule<Iterator,

91  std::vector<expression>(var\_origin),

92  whitespace\_grammar<Iterator> >

93  opt\_dims\_r;

94

95  boost::spirit::qi::rule<Iterator,

96  ordered\_var\_decl(var\_origin),

97  whitespace\_grammar<Iterator> >

98  ordered\_decl\_r;

99

100  boost::spirit::qi::rule<Iterator,

101  positive\_ordered\_var\_decl(var\_origin),

102  whitespace\_grammar<Iterator> >

103  positive\_ordered\_decl\_r;

104

105  boost::spirit::qi::rule<Iterator,

106  range(var\_origin),

107  whitespace\_grammar<Iterator> >

108  range\_brackets\_double\_r;

109

110  boost::spirit::qi::rule<Iterator,

111  range(var\_origin),

112  whitespace\_grammar<Iterator> >

113  range\_brackets\_int\_r;

114

115  boost::spirit::qi::rule<Iterator,

116  row\_vector\_var\_decl(var\_origin),

117  whitespace\_grammar<Iterator> >

118  row\_vector\_decl\_r;

119

120  boost::spirit::qi::rule<Iterator,

121  unit\_vector\_var\_decl(var\_origin),

122  whitespace\_grammar<Iterator> >

123  unit\_vector\_decl\_r;

124

125  boost::spirit::qi::rule<Iterator,

126  simplex\_var\_decl(var\_origin),

127  whitespace\_grammar<Iterator> >

128  simplex\_decl\_r;

129

130  boost::spirit::qi::rule<Iterator,

131  vector\_var\_decl(var\_origin),

132  whitespace\_grammar<Iterator> >

133  vector\_decl\_r;

134

135  boost::spirit::qi::rule<Iterator,

136  boost::spirit::qi::locals<bool>,

137  var\_decl(bool,var\_origin),

138  whitespace\_grammar<Iterator> >

139  var\_decl\_r;

140

141  boost::spirit::qi::rule<Iterator,

142  boost::spirit::qi::locals<bool>,

143  std::vector<var\_decl>(bool,var\_origin),

144  whitespace\_grammar<Iterator> >

145  var\_decls\_r;

146

147

148  };

149

150  }

151 }

152

153 #endif

stan::gm::var\_decls\_grammar::identifier\_name\_r

boost::spirit::qi::rule< Iterator, std::string(), whitespace\_grammar< Iterator > > identifier\_name\_r

**Definition:** var\_decls\_grammar.hpp:78

stan::gm::corr\_matrix\_var\_decl

**Definition:** ast.hpp:563

stan::gm::var\_decls\_grammar::matrix\_decl\_r

boost::spirit::qi::rule< Iterator, matrix\_var\_decl(var\_origin), whitespace\_grammar< Iterator > > matrix\_decl\_r

**Definition:** var\_decls\_grammar.hpp:88

stan::gm::double\_var\_decl

**Definition:** ast.hpp:464

stan::gm::var\_decls\_grammar::var\_decls\_grammar

var\_decls\_grammar(variable\_map &var\_map, std::stringstream &error\_msgs)

**Definition:** var\_decls\_grammar\_def.hpp:653

stan::gm::var\_decls\_grammar::row\_vector\_decl\_r

boost::spirit::qi::rule< Iterator, row\_vector\_var\_decl(var\_origin), whitespace\_grammar< Iterator > > row\_vector\_decl\_r

**Definition:** var\_decls\_grammar.hpp:118

expression07\_grammar.hpp

stan::gm::row\_vector\_var\_decl

**Definition:** ast.hpp:514

stan::gm::range

**Definition:** ast.hpp:401

stan::gm::ordered\_var\_decl

**Definition:** ast.hpp:488

expression\_grammar.hpp

stan::gm::expression\_grammar

**Definition:** expression07\_grammar.hpp:23

stan::gm::var\_decls\_grammar::cholesky\_factor\_decl\_r

boost::spirit::qi::rule< Iterator, cholesky\_factor\_var\_decl(var\_origin), whitespace\_grammar< Iterator > > cholesky\_factor\_decl\_r

**Definition:** var\_decls\_grammar.hpp:43

stan::gm::var\_decls\_grammar::dims\_r

boost::spirit::qi::rule< Iterator, std::vector< expression >var\_origin), whitespace\_grammar< Iterator > > dims\_r

**Definition:** var\_decls\_grammar.hpp:63

stan::gm::var\_decls\_grammar::expression07\_g

expression07\_grammar< Iterator > expression07\_g

**Definition:** var\_decls\_grammar.hpp:36

stan::gm::var\_decls\_grammar

**Definition:** var\_decls\_grammar.hpp:20

stan::gm::cov\_matrix\_var\_decl

**Definition:** ast.hpp:554

stan::gm::var\_decls\_grammar::unit\_vector\_decl\_r

boost::spirit::qi::rule< Iterator, unit\_vector\_var\_decl(var\_origin), whitespace\_grammar< Iterator > > unit\_vector\_decl\_r

**Definition:** var\_decls\_grammar.hpp:123

stan::gm::var\_decls\_grammar::vector\_decl\_r

boost::spirit::qi::rule< Iterator, vector\_var\_decl(var\_origin), whitespace\_grammar< Iterator > > vector\_decl\_r

**Definition:** var\_decls\_grammar.hpp:133

stan::gm::var\_decl

**Definition:** ast.hpp:592

stan::gm::cholesky\_factor\_var\_decl

**Definition:** ast.hpp:536

stan::gm::var\_decls\_grammar::corr\_matrix\_decl\_r

boost::spirit::qi::rule< Iterator, corr\_matrix\_var\_decl(var\_origin), whitespace\_grammar< Iterator > > corr\_matrix\_decl\_r

**Definition:** var\_decls\_grammar.hpp:53

stan::gm::positive\_ordered\_var\_decl

**Definition:** ast.hpp:496

stan::gm::variable\_map

**Definition:** ast.hpp:439

stan::gm::var\_decls\_grammar::identifier\_r

boost::spirit::qi::rule< Iterator, std::string(), whitespace\_grammar< Iterator > > identifier\_r

**Definition:** var\_decls\_grammar.hpp:73

stan::gm::var\_decls\_grammar::var\_decls\_r

boost::spirit::qi::rule< Iterator, boost::spirit::qi::locals< bool >, std::vector< var\_decl >bool, var\_origin), whitespace\_grammar< Iterator > > var\_decls\_r

**Definition:** var\_decls\_grammar.hpp:145

stan::gm::var\_decls\_grammar::opt\_dims\_r

boost::spirit::qi::rule< Iterator, std::vector< expression >var\_origin), whitespace\_grammar< Iterator > > opt\_dims\_r

**Definition:** var\_decls\_grammar.hpp:93

stan::gm::var\_decls\_grammar::cholesky\_corr\_decl\_r

boost::spirit::qi::rule< Iterator, cholesky\_corr\_var\_decl(var\_origin), whitespace\_grammar< Iterator > > cholesky\_corr\_decl\_r

**Definition:** var\_decls\_grammar.hpp:48

stan::gm::var\_decls\_grammar::ordered\_decl\_r

boost::spirit::qi::rule< Iterator, ordered\_var\_decl(var\_origin), whitespace\_grammar< Iterator > > ordered\_decl\_r

**Definition:** var\_decls\_grammar.hpp:98

stan::gm::var\_decls\_grammar::double\_decl\_r

boost::spirit::qi::rule< Iterator, double\_var\_decl(var\_origin), whitespace\_grammar< Iterator > > double\_decl\_r

**Definition:** var\_decls\_grammar.hpp:68

stan::gm::var\_decls\_grammar::var\_decl\_r

boost::spirit::qi::rule< Iterator, boost::spirit::qi::locals< bool >, var\_decl(bool, var\_origin), whitespace\_grammar< Iterator > > var\_decl\_r

**Definition:** var\_decls\_grammar.hpp:139

stan::gm::matrix\_var\_decl

**Definition:** ast.hpp:524

stan::gm::var\_decls\_grammar::range\_brackets\_double\_r

boost::spirit::qi::rule< Iterator, range(var\_origin), whitespace\_grammar< Iterator > > range\_brackets\_double\_r

**Definition:** var\_decls\_grammar.hpp:108

stan::gm::simplex\_var\_decl

**Definition:** ast.hpp:480

stan::gm::cholesky\_corr\_var\_decl

**Definition:** ast.hpp:546

stan::gm::int\_var\_decl

**Definition:** ast.hpp:455

ast.hpp

stan::gm::var\_decls\_grammar::range\_brackets\_int\_r

boost::spirit::qi::rule< Iterator, range(var\_origin), whitespace\_grammar< Iterator > > range\_brackets\_int\_r

**Definition:** var\_decls\_grammar.hpp:113

stan::gm::var\_decls\_grammar::positive\_ordered\_decl\_r

boost::spirit::qi::rule< Iterator, positive\_ordered\_var\_decl(var\_origin), whitespace\_grammar< Iterator > > positive\_ordered\_decl\_r

**Definition:** var\_decls\_grammar.hpp:103

stan::gm::whitespace\_grammar

**Definition:** whitespace\_grammar.hpp:11

stan::gm::unit\_vector\_var\_decl

**Definition:** ast.hpp:472

stan::gm::var\_decls\_grammar::error\_msgs\_

std::stringstream & error\_msgs\_

**Definition:** var\_decls\_grammar.hpp:31

whitespace\_grammar.hpp

stan::gm::var\_decls\_grammar::cov\_matrix\_decl\_r

boost::spirit::qi::rule< Iterator, cov\_matrix\_var\_decl(var\_origin), whitespace\_grammar< Iterator > > cov\_matrix\_decl\_r

**Definition:** var\_decls\_grammar.hpp:58

stan::gm::var\_decls\_grammar::int\_decl\_r

boost::spirit::qi::rule< Iterator, int\_var\_decl(var\_origin), whitespace\_grammar< Iterator > > int\_decl\_r

**Definition:** var\_decls\_grammar.hpp:83

stan::gm::var\_decls\_grammar::expression\_g

expression\_grammar< Iterator > expression\_g

**Definition:** var\_decls\_grammar.hpp:34

stan::gm::vector\_var\_decl

**Definition:** ast.hpp:504

stan::gm::expression07\_grammar

**Definition:** expression07\_grammar.hpp:26

stan::gm::var\_decls\_grammar::simplex\_decl\_r

boost::spirit::qi::rule< Iterator, simplex\_var\_decl(var\_origin), whitespace\_grammar< Iterator > > simplex\_decl\_r

**Definition:** var\_decls\_grammar.hpp:128

stan::gm::var\_decls\_grammar::var\_map\_

variable\_map & var\_map\_

**Definition:** var\_decls\_grammar.hpp:30

stan::gm::var\_origin

int var\_origin

**Definition:** ast.hpp:411


---

[ Stan Home Page ]

*© 2011–2014,
Stan Development Team.*
